# Editorial: Kappa opioid receptors revealed: Disentangling the pharmacology to open up new therapeutic strategies

**DOI:** 10.3389/fphar.2022.973780

**Published:** 2022-08-12

**Authors:** Wendy Margaret Walwyn, Marta Valenza

**Affiliations:** ^1^ Jane & Terry Semel Institute for Neuroscience and Human Behavior, Los Angeles, CA, United States; ^2^ Department of Physiology and Pharmacology “Vittorio Erspamer”, Sapienza University of Rome, Rome, Italy

**Keywords:** addiction, biased agonism, dynorphins, G-protein, Kappa opioid receptor OR KOR OR KOPr OR KOP, opioids, pain, stress

Three families of opioid peptides, beta-endorphin, enkephalins, and dynorphins, and three families of receptors, mu (MOR), delta (DOR), and kappa (KOR), constitute the endogenous opioid system. Opioid receptors are found throughout the central and peripheral nervous systems in both pre- and post-synaptic neurons modulating neurotransmitter release (Marvizon et al., 2015). Their differential expression and location determine the variety of effects that they mediate, as they are typically concentrated in circuits involved in pain modulation, autonomic control, mood, reward, and stress response. Discriminating the contribution of each component to opioid behavioral effects have been complicated by the close homology among receptor isoforms, and the variety of opioid peptides formed by precursors, with differing affinity for each receptor subtype (Valentino and Volkow, 2018). The finding that opioid receptors may assemble and operate as heteromers, not just with opioid isoforms but also with other receptor classes, adds a new level of complexity to the equation, but it also opens up new avenues for manipulating their function and behavioral repercussions (Valentino and Volkow, 2018; Valenza et al., 2020a). The present Research Topic focuses on the kappa opioid receptor (KOR) and its pharmacology, since growing evidence has underlined its potential as a therapeutic target for several neurologic and psychiatric conditions, from pain management to addiction, from itch to stress-related disorders.

Being G-coupled inhibitory protein receptors (G_i_/G_0_), endogenous ligands or exogenous agonists that activate KOR receptors generally suppress neuronal activity or neurochemical release. A range of intracellular events mediate these inhibitory effects, which may include adenyl cyclase inhibition, reduced voltage-gated calcium channel opening, and potassium current stimulation through different channels, including G-protein inwardly rectifying potassium channels (GIRKs) (Marvizon et al., 2015). Given the variety of signaling pathways that it mediates, the KOR and its endogenous ligands, the dynorphins, play a pivotal role in a wide range of physiological functions. The huge technical advancements we have seen in recent years have been invaluable to begin disentangling the pharmacology of KOR. As a result, two KOR agonists have very recently reached clinical approval as antipruritic agents (difelikefalin in US and EU, and nalfurafine in Japan). However, there is still no definitive therapy for depression, addiction, post-traumatic stress disorder, for which KOR ligands show therapeutic potential. As such, the present article collection has stimulated scientists to report new studies on how dynorphins/KOR signaling modulates neurotransmission and affects behaviors.

The KOR ligands synthetized so far have distinct pharmacological properties, as agonists, partial agonists, biased agonists, and antagonists (the last with both long and short half-lives). The most known KOR agonist effects are the antinociceptive and anti-pruritic ones, but they are not limited to them; this Research Topic actually includes two comprehensive analyses of the literature concerning the potential utility of KOR ligands as therapeutics (Dalefield et al.; Leconte et al.). Unfortunately, KOR agonists clinical use has been hampered by their adverse effects, as dysphoria and psychotic symptoms, motor incoordination and sedation. It has been proposed that a mechanism involving the activation of intracellular β-arrestin/MAPK pathway mediates such unwanted effects (Bruchas and Chavkin, 2010). However, Liu-Chen’s group here challenges this view by testing the relationship of β-arrestin recruitment to the aversive properties of four different KOR agonists (U50,488H, methoxymethyl salvinorin B, nalfurafine, and 3-deoxynalfurafine) presenting compelling data that show KOR agonist-induced aversive effects in male mice to be unrelated to KOR phosphorylation and internalization (Chen et al.). Further, Sturaro et al. present two novel agonists (PWT2-Dyn A and Dyn A-palmitic) confronting their properties to other well-known KOR ligands to classify each ligand action as full, partial, inverse agonist or neutral antagonists using a battery of assays. Of note, they designate Dyn A-palmitic as a new agonist biased towards G-protein signaling. Further studies are needed to elucidate if it exerts analgesic properties *in vivo* with no side effects.

KOR-mediated signaling regulates many aspects of the mesocorticolimbic dopamine system, contributing to the negative affective states occurring in a variety of neuropsychiatric disorders as well as in chronic pain (Carlezon and Krystal, 2016; Tejeda and Bonci, 2019). Both KOR expression and dynorphins tone can be up-regulated in several brain areas and pathways by stress exposure, chronic pain or by a history of drug abuse (McLaughlin et al., 2003; Valenza et al., 2016; Liu et al., 2019). In this context, KOR antagonism has been proposed as therapeutic approach for stress-related conditions and in cases of comorbidity with addiction (Leconte et al.). However, most of the knowledge we have accumulated so far on KOR blockade is based on studies employing drugs with unusually long duration of action and unclear pharmacokinetics. Despite this complicates both interpretation of results and translation of preclinical findings into the clinic, such long-acting KOR antagonists can still be useful tools to unravel the pharmacology of KOR. Indeed here, using norBNI, a study from the Neugebauer’s group provides new insights into the function of amygdalar KOR, demonstrating that KOR regulates rodent pain and aversive-like behaviors by inhibiting corticotropin releasing factor neurons of the central amygdala in a rat model of functional pain syndrome (Yakhnitsa et al.). A new generation of KOR antagonists with rapid onset and shorter duration of action can provide useful data that may be of interest for medication development. In this context, recent reports have shown that some novel short-acting KOR antagonists can attenuate or even block behaviors that are relevant to cocaine, nicotine and ethanol addiction in rats (Jackson et al., 2015; Valenza et al., 2017; Domi et al., 2018; Valenza et al., 2020b). Baynard et al. shows here that a congener molecule (LY2795050) can produce very rapid anti-immobility effect in an open space swim test as a model of acute stress exposure. Despite the large amount of preclinical evidence gathered to date and the fact that some molecules have already reached the clinical trial stage, no KOR antagonist has yet been approved for therapy. Thus, further research efforts are needed.

The plethora of drugs synthetized that can modulate the KOR signaling is invaluable to unveil the complex pharmacology of this receptor and a further understanding of its molecular and behavioral pharmacology is essential. The collection of articles here presented perfectly fits this scope and contributes to advance the knowledge in the field.
